# Thalassemia in the United Arab Emirates: Why it can be prevented but not eradicated

**DOI:** 10.1371/journal.pone.0170485

**Published:** 2017-01-30

**Authors:** Sehjeong Kim, Abdessamad Tridane

**Affiliations:** Department of Mathematical Sciences/United Arab Emirates University, Al Ain, Abu Dhabi, United Arab Emirates; University of Naples Federico II, ITALY

## Abstract

Thalassemia is a genetic blood disorder that causes abnormal hemoglobin. Hemoglobin is a protein in red blood cells that carries oxygen and is made of two proteins from four *α*-globin genes and two *β*-globin genes. A defect in one or more of these genes causes thalassemia. The treatment of thalassemia mostly depends on life-long blood transfusions and removal of excessive iron from the blood stream. Such tremendous blood consumption puts pressure on the national blood stock in many countries. In particular, in the United Arab Emirates (UAE), various forms of thalassemia prevention have been used and hence, the substantial reduction of the thalassemia major population has been achieved. However, the thalassemia carrier population still remains high, which leads to the potential increase in the thalassemia major population through carrier-carrier marriages. In this work, we investigate the long-term impact and efficacy of thalassemia prevention measures via mathematical modeling at a population level. To our best knowledge, this type of assessment has not been done before and there is no mathematical model that has investigated such a problem for thalassemia or any blood disorders at a population level. By using UAE data, we perform numerical simulations of our model and conduct sensitivity analysis of parameter values to see which parameter values affect most the dynamics of our model. We discover that the prevention measures can contribute to reduce the prevalence of the disease only in the short term but not eradicate the disease in the long term.

## Introduction

Thalassemia is a genetic blood disorder in which the body makes an abnormal form of hemoglobin, the protein in red blood cells that carries oxygen. Hemoglobin is made of two proteins from four *α*-globin genes and two *β*-globin genes. There are two types of thalassemia: *α*-thalassemia and *β*-thalassemia depending on which globin genes are defected. *α*-thalassemia occurs when one or more of the four *α*-globin genes are missing, damaged or changed. *β*-thalassemia occurs when both *β*-globin genes are affected [1–4]. Thalassemia major occurs when a child inherits two defected globin genes, one from each parent. Thalassemia minor occurs when a child inherits one defected globin gene from only one parent. People with thalassemia minor usually do not show symptoms and can live a normal life without treatment. Also, a person with thalassemie minor is often called a thalassemia carrier, or simply just a carrier. Thus, for example, if a child is diagnosed with *α*-thalassemia major, the child inherited two defected *α*-globin genes from both parents. If a child is a *β*-thalassemia carrier, the child inherited one defected *β*-globin gene from one of his or her parents. Thalassemia major usually causes a chronic, lifelong anemia that begins in early childhood and often must be treated with frequent blood transfusions due to the deformity of red blood cells. Because of the requirement of life-long blood transfusion as a treatment protocol, thalassemia treatment requires a huge amount of national blood stock, which could put pressure on the use of blood for other types of treatments. Moreover, thalassemia treatment often results in significant complications including iron overload, bone deformities and cardiovascular illness.

Thus, thalassemia has been a burden on the healthcare systems of many countries in the Mediterranean area, the Middle Eastern and North African (MENA) region, Transcaucasia, Central Asia, the Indian subcontinent, and Southeast Asia [4, 5]. Due to the migration of people from these regions, thalassemia populations have become a public health concern even in North America [5]. Today, worldwide, 56,000 conceptions cause thalassemia; of these, approximately 30,000 are affected by *β*-thalassemia and 3,500 succumb perinatally from the hydrops fetalis syndrome, a type of *α*-thalassemia. It has also been estimated that, worldwide, 9 million thalassemia carrier women become pregnant annually and 1.33 million pregnancies are at risk for a thalassemia major condition [6, 7]. In particular, the MENA region shows a high prevalence of thalassemia major and carrier populations due to a consanguineous marriage tradition [8, 9]. A consanguineous marriage can be defined as a marriage between two individuals who are related as second cousins or closer with the inbreeding coefficient equal to or higher than 0.0156 [10], where the inbreeding coefficient represents a measure of the proportion of loci at which the offspring of a consanguineous marriage is expected to inherit identical gene copies from both parents. It has been found that consanguineous marriages lead to the increased expression of autosomal recessive disorders [11–14].

Among the countries in the MENA region, the United Arab Emirtes (UAE) with around a 50% consanguineous marriage rate as shown in Table 1 [15–18] has a substantial prevalence of thalassemia carrier. This rate has been increasing and the explanation for such an increase is not yet known [8]. The high prevalence of a thalassemia carrier population is a public health concern since there will be a 25% chance of having a thalassemia major child if a carrier-carrier marriage occurs, leading to a potential increase in the thalassemia major population. In addition, the UAE has the most various heterozygote of *β*-thalassemia [8, 15]. The UAE government has been expending tremendous effort to raise public awareness and identify the thalassemia carrier population. In 2008, the UAE government launched a nationwide campaign to promote premarital screening. Since 2012, premarital screening has been mandatory for all about-to-marry couples [19]. Prematrital screening offers a non-directive genetic counseling to at-risk couples [20]. At-risk couples who are carrier-carrier couples receive information about the risk of having a thalassemia child and options of prenatal and neonatal diagnosis and preimplantation diagnosis. However, the final decision to get married lies on them and many carrier-carrier couples choose to marry anyway. Also, it is worth mentioning that the termination of pregnancy regarding thalassemia is not practiced as a solution for the prevention of thalassemia in the UAE. Today, the number of affected births has been almost halved compared to the time before the introduction of prevention [21]. However, continued public awareness, promotion of prevention programs, and a centralized and cooperative national genetic service are still needed for thalassemia prevention in the UAE [8].

**Table 1:** Consanguineous marriage rates and prevalence of thalassemia carrier in the MENA region.

Country	Consanguineous Marriage (%)	Inbreeding Coefficient	*β*-thalassemia carrier (%)	*α*-thalassemia carrier (%)
Bahrain	31.8–44.5	0.0152–0.0166	2.9	24.2
Egypt	29	0.0101	4.5	N/A
Jordan	25.6–52.9	0.0142–0.0284	3–5.9	2–3.5
Kuwait	54.3	0.047	N/A	5–10
Saudi Arabia	41.4–51.7	0.0196–0.0312	1–15	5–10
UAE	50.5	0.0222	8.5	49

The motive behind all these efforts toward thalassemia prevention is based on the two hypotheses:

(H1) Premarital screening will prevent the intermarriage in the carrier population;
(H2) In addition to premarital screening, if young adults are educated about thalassemia, marriage among the carrier populations will be prevented.

The two hypotheses have been made by many governments that need to manage thalassemia in their countries. The baseline expectation of the hypotheses is the reduction of the thalassmia major population and eventual elimination of thalassemia from the population. Nowadays, the UAE as many other countries do celebrates an almost-thalassemia-free status due to various prevention methods [22]. However, as a relatively young country with a high proportion of working population [19], the UAE’s population growth will continue and hence the thalassemia carrier population will likewise grow. This implies that a high prevalence of thalassemia carrier population in the UAE will continue. As the carrier population continues to marry each other, thalassemia cases are highly likely to reappear. In this paper, we use a mathematical model and computer simulations with UAE population data to test the two hypotheses and investigate the possibility that thalassemia in the UAE can be eradicated via thalassemia prevention methods. We would also like to make the case that for the foreseeable future the UAE government needs to continue its attention to the thalassemia prevention program at existing levels.

## Methods

### Model Formulation and Assumptions

With a mathematical model using UAE population data, we investigate whether thalassemia prevention methods such as premarital screening and education can achieve the eradication of thalassemia in the long term. Here, we would like to emphasize that education includes all types of possible genetic counseling and efforts toward public awareness of thalassemia by health authorities in the UAE.

Our approach is to use a *compartment model* that divides the whole population into several subgroups in the UAE. Then, we describe the interactions among the subgroups via differential equations. In our model, we classify the subgroup populations into the following three age groups, three genetic groups, two screening groups, and two thalassemia education groups as

- Children: Aged 12 or under in primary school level or lower
  Young adults: Aged 13–24 at or above middle school including college level before marriage
  Adults: Aged 25 or older after college level, marriageable or married;
- Normal: Inherited no thalassemia genes from parents
  Carrier: Inherited one thalassemia gene from parents
  Major: Inherited two thalassemia genes from parents;
- Screened Normal and Screened Carrier: Subgroups who are screened and whose genetic status is verified;
- Educated and Not educated: Subgroups who are educated about thalassemia or not.

The young adult group includes adolescents aged 13–18 who can receive education about thalassemia and college students or older aged 19–24 who may have education about thalassemia and are likely to get premarital screening. In other words, people in this group can receive education about thalassemia and need to take premarital screening if they are going to marry. These classifications can overlap. For example, an individual can be classified as a carrier educated young adult if he or she is around twenty five years old, not married and screened as a carrier who received the education about thalassemia. Since thalassemia is a genetic disease passed to the next generation through marriage, it is natural to consider both genders in all classes except the married class. We consider a married couple as one unit. Then, we assume the following:

1. Since the average marriage age is 24.5 in the UAE [23], premarital screening for thalassemia is given to those in the young adult group who are about to marry.
2. Thalassemia awareness education is given to the young adult population and genetic counseling is given to marriageable couples.
3. People who receive premarital screening or thalassemia awareness education can reconsider their marriage decision.
4. The thalassemia major class has a very low chance of marriage due to prolonged treatments. Hence, the marriage rate of the thalassemia major class is negligible.
5. Except for the thalassemia major class, all other classes can marry.
6. The chances of marriages among single males and females are
  - normal male × normal female
  - normal male × carrier female or carrier male × normal female
  - carrier male × carrier female

We define each population subgroup with the variable names as shown in Table 2. For the model, the following parameter values are used based on the UAE population data shown in Table 3. With the variables and parameters, we describe the dynamics of all subgroups (compartments) via differential equations as follows. For example, the dynamics of children (boys) and thalassemia male can be written as

$$\begin{array}{c} \begin{array}{ccc} \frac{dG_{M}}{dt} & = & \;\text{Birth}\;-\;\text{Diagnosed}\;\text{thalassemia}\;\text{boys}\\ & & \;-\text{Growth to young adult male}-\text{Death}\\ & = & b_{M}U-\zeta \eta_{T}^{M}G_{M}-\left(1-\zeta \eta_{T}^{M}\right)\gamma_{M}G_{M}-d_{M}^{G}G_{M}, \end{array} \end{array}$$ (1)

$$\begin{array}{c} \begin{array}{ccc} \frac{dT_{M}}{dt} & = & \;\text{Diagnosed}\;\text{thalassemia}\;\text{boys}\\ & & \;-\text{Reduction of thalassemia due to education}\\ & & \;-\text{Death}\\ & = & \zeta \eta_{T}^{M}G_{M}-\left(\frac{\varepsilon \left(1-\zeta \eta_{T}^{M}\right)\gamma_{M}G_{M}}{\gamma_{M}G_{M}+\gamma_{F}G_{F}}\right)T_{M}-d_{T}T_{M}, \end{array} \end{array}$$ (2)

**Fig 1:**
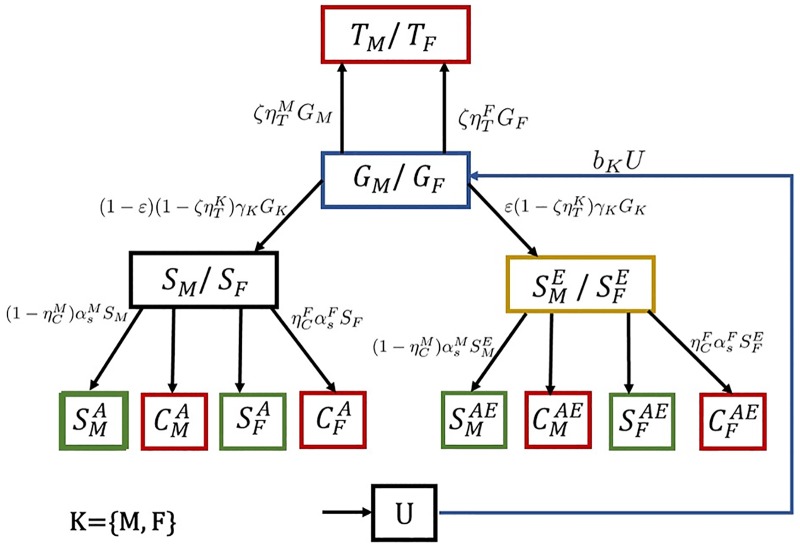
Schema of population dynamics of the mathematical model. Each box represents a population subgroup, i.e. compartment. The arrows indicate how the population in each compartment could move from one compartment to another via our mathematical model. The variable names are found in Table 2 and the mathematical equations on the arrows can be found in S1 Material. For example, *b*_*K*_ *U* means newborn boys or girls from married coupled and hence they are moved to the children compartments *G*_*M*_ or *G*_*F*_.

The dynamics of single normal young adult males who are either educated or not educated about thalassemia can be described as

$$\begin{array}{c} \begin{array}{ccc} \frac{dS_{M}^{E}}{dt} & = & \;\text{Educated}\;\text{young}\;\text{adult}\;\text{males}\\ & & \;-\;\text{Screened}\;\text{marriageable}\;\text{males}\;-\;\text{Death}\\ & = & \varepsilon \left(1-\zeta \eta_{T}^{M}\right)\gamma_{M}G_{M}-\alpha_{s}^{M}S_{M}^{E}-d_{M}S_{M}^{E}, \end{array} \end{array}$$ (3)

$$\begin{array}{c} \begin{array}{ccc} \frac{dS_{M}}{dt} & = & \;\text{Uneducated}\;\text{young}\;\text{adult}\;\text{males}\\ & & \;-\;\text{Screened}\;\text{marriageable}\;\text{males}\;-\;\text{Death}\\ & = & \left(1-\varepsilon \right)\left(1-\zeta \eta_{T}^{M}\right)\gamma_{M}G_{M}-\alpha_{s}^{M}S_{M}-d_{M}S_{M}, \end{array} \end{array}$$ (4)

where premarital screening is given to marriageable single young adult males. The dynamics of educated single and carrier female adults is given by

$$\begin{array}{c} \begin{array}{ccc} \frac{dS_{F}^{AE}}{dt} & = & \;\text{Screened}\;\text{educated}\;\text{normal}\;\text{adult}\;\text{females}\\ & & \;-\;\text{Marriage}\;\text{with}\;\text{male}\;\text{marriageable}\;\text{subgroups}\\ & & \;-\;\text{Death}\\ & = & \left(1-\eta_{C}^{F}\right)\alpha_{s}^{F}S_{F}^{E}\\ & & \;-\alpha_{F}S_{F}^{AE}\frac{\left(S_{M}^{AE}+C_{M}^{AE}+S_{M}^{A}+C_{M}^{A}\right)}{N^{A}}\\ & & \;-d_{M}S_{M}^{AE}, \end{array} \end{array}$$ (5)

$$\begin{array}{c} \begin{array}{ccc} \frac{dC_{F}^{AE}}{dt} & = & \;\text{Screened}\;\text{educated}\;\text{adult}\;\text{carrier}\;\text{females}\\ & & \;-\;\text{Marriage}\;\text{with}\;\text{male}\;\text{marriageable}\;\text{subgroups}\\ & & \;+\;\text{Reconsideration}\;\text{of}\;\text{marriage}\;\text{with}\;\text{carrier}\;\text{males}\;\\ & & \;-\;\text{Death}\\ & = & \eta_{C}^{F}\alpha_{s}^{F}S_{F}^{E}-\alpha_{F}C_{F}^{AE}\frac{\left(S_{M}^{AE}+C_{M}^{AE}+S_{M}^{A}+C_{M}^{A}\right)}{N^{A}}\\ & & +{\tilde{\nu }}_{F}\alpha_{F}C_{F}^{AE}\frac{\left(C_{M}^{A}+C_{M}^{AE}\right)}{N^{A}}-d_{F}C_{F}^{AE}, \end{array} \end{array}$$ (6)

where the reconsideration of marriage of the carrier females with carrier males means the dissolution of the marriage as an outcome of the education and premarital screening. The schema of the population dynamics is shown in Fig 1. For the full scope of the mathematical model, see S1 Material.

**Table 2:** Subgroup variable names.

Subgroup Variable	Characteristics
*G*_*M*_, *G*_*F*_	Children: Boys and girls aged 0–12
*T*_*M*_, *T*_*F*_	Thalassemia major population
*S*_*M*_, *S*_*F*_	Normal singles uneducated about thalassemia: Male and female young adults aged 13–24
$S_{M}^{E},$ $S_{F}^{E}$	Normal singles educated about thalassemia: Male and female young adults aged 13–24
$S_{M}^{AE},$ $S_{F}^{AE}$	Normal singles educated about thalassemia: Male and female adults screened and marriageable aged 25 or older
$C_{M}^{AE},$ $C_{F}^{AE}$	Carrier singles educated about thalassemia: Male and female adults screened and marriageable aged 25 or older
$S_{M}^{A},$ $S_{F}^{A}$	Normal singles not educated about thalassemia: Male and female adults screened and marriageable aged 25 or older
$C_{M}^{A},$ $C_{F}^{A}$	Carrier singles not educated about thalassemia: Male and female adults screened and marriageable aged 25 or older
*U*	Married (United) population

**Table 3:** Literature based and estimated parameter values.

Parameters	Name	Male	Female	References
*b*_*M*_ | *b*_*F*_	Birth rates†	0.0363	0.0352	[24]
*γ*_*M*_ | *γ*_*F*_	Proportion of young adults†	0.0433	0.0447	[19]
*α*_*M*_ | *α*_*F*_	Marriage rates†	0.02	0.0172	[24]
$\alpha_{S}^{M}$ | $\alpha_{S}^{F}$	Premarital screening rates†	0.0413	0.0335	[24]
$\eta_{C}^{M}$ | $\eta_{C}^{F}$	Thalassemia carrier detection rates†	0.0833	0.0833	[16]
$\eta_{T}^{M}$ | $\eta_{T}^{F}$	Thalassemia detection adjusting rates†	0.15	0.144	[25]
*d*_*M*_ | *d*_*F*_	Normal death rates†	0.003	0.0019	[24]
$d_{M}^{G}$ | $d_{F}^{G}$	Child mortality rates†	0.014	0.008	[23]
*d*_*T*_	Death rate due to thalassemia†	0.016	0.016	[1]
*ν*_*M*_ | *ν*_*F*_	Marriage reconsideration rates (MRR)	30%	30%	−
${\tilde{\nu }}_{M}$ | ${\tilde{\nu }}_{F}$	MRR of educated singles	50%	50%	−
*ε*	Education rate of marriageable singles	20%	20%	−

^†^ The unit of parameter values except *ν*_*M*_, *ν*_*F*_, ${\tilde{\nu }}_{M},$ ${\tilde{\nu }}_{F},$ and *ε* is per person. Since marriage reconsideration rate or education rate has not been found, we assign values for simulation purpose in our study.

Then, the mathematical model explains the evolution of the population subgroups in $(G_{M},G_{F},T_{M},T_{F},S_{M},S_{M}^{AE},C_{M}^{AE},S_{M}^{A},C_{M}^{A},S_{F},S_{F}^{AE},C_{F}^{AE},S_{F}^{A},C_{F}^{A},U).$ To see the effect of the education and premarital screening on the eradication of thalassemia in the long term, we will study the sensitivity of parameters and two steady states called equilibrium points such as

(i) Type I Thalassemia free equilibrium point, i.e., *T*_*M*_ = *T*_*F*_ = 0, $C_{M}^{A}=C_{M}^{AE}=0,$ and $C_{F}^{A}=C_{F}^{AE}=0;$
(ii) Type II Thalassemia major free only equilibrium point, i.e., *T*_*M*_ = *T*_*F*_ = 0 only and $C_{M}^{A},$ $C_{M}^{AE},$ $C_{F}^{A},$ $C_{F}^{AE}$ are not necessarily zero

in the following section via a mathematical analysis and computer simulations. All our simulations are generated with R software [26], and the sensitivity analysis with Flexible Modelling Environment (FME) package [27].

## Results

First, we show that the considered model is well posed in S1 Material. Second, we investigate the stability of two possible disease free equilibrium points, Types I and II equilibrium points. Here, Type I equilibrium point implies the eventual extinction of thalassemia major and carrier populations, and Type II equilibrium point indicates the extinction of only thalassemia major population in the future. We focus on these equilibrium points only because they are the aim of any thalassemia control to eradicate the disease.

**Fig 2:**
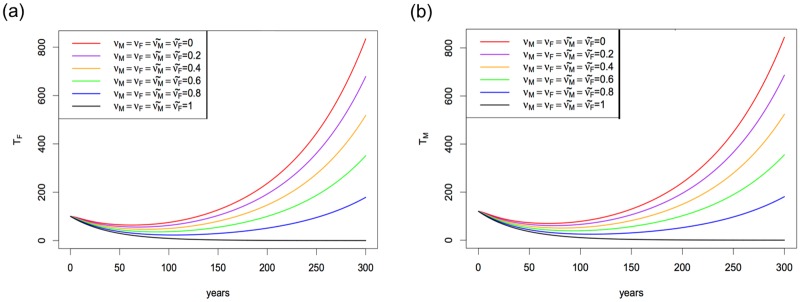
Simulation results of thalassemia major population in both genders with different level of marriage reconsideration rates. (a) Thalassemia female *T*_*F*_ with different marriage reconsideration rates. (b) Thalassemia male *T*_*M*_ with different marriage reconsideration rates. *ν*_*K*_ and ${\tilde{\nu }}_{K}$ are the marriage reconsideration rates of educated and uneducated carrier populations for *K* = {*M*, *F*}. *ν*_*K*_ = 0 ($\tilde{\nu_{K}}=0$) means all of uneducated (educated) carrier males or females do not give up their marriage decision, whereas *ν*_*K*_ = 1 ($\tilde{\nu_{K}}=1$) means all of uneducated (educated) carrier males or females do give up their marriage decision due to screening and education. The education and premarital screening rates are as in Table 3.

Through the analysis of our mathematical model, we verified that Types I and II equilibrium points are both *unstable* under the education and premarital screening factors (see the proof in S1 Material). That is, although we may be able to push a given thalassemia status in the UAE toward Type I or Type II equilibrium status by premarital screening and education factors, we will never achieve thalassemia free or thalassemia major free only status in the long term. Thus, thalassemia prevention via premarital screening and education is possible but not effective enough to achieve thalassemia free status. However, this result does not mean that the education and premarital screening are not effective at all. Since these two prevention measures could send a message about the risk of carrier-carrier marriage, i.e. 25% chance of having thalassemia major children, the influence of the two prevention measures should be considered in the marriage decision of carrier-carrier couples. For fixed education and premarital screening rates as in Table 3, we vary the marriage reconsideration rates from 0% to 100% with the initial data $G_{M}^{o}=16,040,$ $G_{F}^{o}=15,350,$ $T_{M}^{o}=120,$ $T_{F}^{o}=100,$ $S_{M}^{o}=100,000,$ $S_{M}^{E^{o}}=20,000,$ $S_{M}^{AE^{o}}=4,500,$ $C_{M}^{AE^{o}}=4,500,$ $S_{M}^{A^{o}}=65,000,$ $C_{M}^{A^{o}}=1,500,$ *S*_*F*^*o*^_ = 100,000, $S_{F}^{E^{o}}=20,000,$ $S_{F}^{AE^{o}}=4,400,$ $C_{F}^{AE^{o}}=3,600,$ $S_{F}^{A^{o}}=55,000,$ $C_{F}^{A^{o}}=1,400,$ and *U*^*o*^ = 15,000. Fig 2 shows that as the marriage reconsideration rates increase, the number of thalassemia major population in both genders decreases. However, this cannot be sustained unless the marriage reconsideration rate of carrier-carrier couples is 100%. Otherwise, the number of thalassemia major cases will increase eventually.

Then, we investigate the sensitivity of the model parameters on variables in order to measure the burden of the disease and the impact of the prevention methods. For each variable, we estimate the sensitivity regarding parameters as they change over time, then eliminate the outliers and plot the variance of the variable in a box plot. For our study, we present the sensitivity analysis of significant parameters with respect to selected variables such as thalassemia and carrier populations.

**Fig 3:**
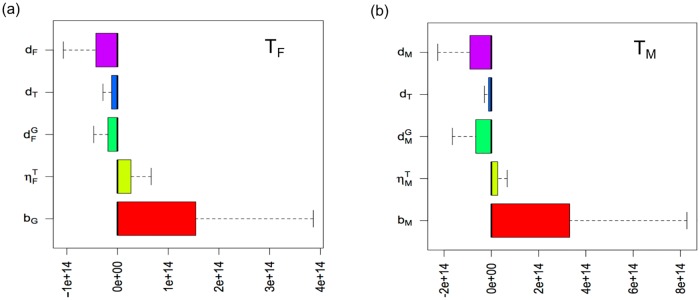
Sensitivity regarding the birth, death, thalassemia diagnosis rates with respect to thalassemia populations. (a) Sensitivity of *b*_*F*_, $\eta_{T}^{F},$ $d_{G}^{F},$ *d*_*T*_ and *d*_*F*_ with respect to *T*_*F*_. (b) Sensitivity of *b*_*M*_, $\eta_{T}^{M},$ $d_{G}^{M},$ *d*_*T*_ and *d*_*M*_ with respect to *T*_*M*_. Note that *b*_*M*_, *b*_*F*_ are birth rates, $d_{G}^{M},$ $d_{G}^{F}$ are child mortality rates, *d*_*M*_, *d*_*F*_ and *d*_*T*_ are normal male and female death rates and thalassemia induced death rates, respectively.

Fig 3 shows that thalassemia major population in both genders tends to increase as birth rates increase. The reason is that marriages between carrier-carrier couples rise as the UAE population grows. Since the carrier population grows as the UAE population grows, marriages between carrier-carrier couples are more likely to increase as well. The increase number of carrier-carrier marriages can increase the potential number of thalassemia major children. Thus, Fig 3 reflects the result of Fig 2. Obviously the death rates (natural, child mortality or thalassemia related) have a negative effect on thalassemia population in both genders. It is interesting to notice that thalassemia major population is more affected by the birth rates than thalassemia detection rates ($\eta_{F}^{T}$ and $\eta_{M}^{T}$) due to the rapid population growth in the UAE.

**Fig 4:**
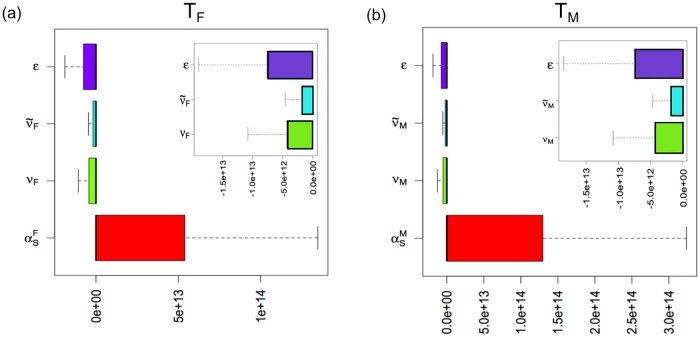
Sensitivity regarding the education, marriage reconsideration and premarital screening rates with respect to thalassemia major population in both genders. (a) Sensitivity of *ϵ*, *ν*_*F*_, ${\tilde{\nu }}_{F},$ and $\alpha_{s}^{F}$ with respect to *T*_*F*_. (b) Sensitivity of *ϵ*, *ν*_*M*_, ${\tilde{\nu }}_{M},$ and $\alpha_{s}^{M}$ with respect to *T*_*M*_. Note that *ε* is the education rate, *ν*_*K*_ and ${\tilde{\nu }}_{K}$ the marriage reconsideration rates of uneducated and educated carrier populations, respectively, and $\alpha_{s}^{K}$ is the premarital screening rate. The inner plots in (a) and (b) are the enlargement of sensitivity regarding *ϵ*, *ν*_*K*_, and ${\tilde{\nu }}_{K},$ where *K* = {*M*, *F*}.

In the inner plots in Fig 4(a) and 4(b), thalassemia major population in both genders decreases as the education rate of single young adults and marriage reconsideration rates of educated and uneducated carrier population increase. Since the education factor indirectly influences the marriage reconsideration of carrier-carrier couples, thalassemia major population in both genders is more sensitive to the education factors than the marriage reconsideration rates. In the outer plots in Fig 4(a) and 4(b), as the rate of premarital screening increases, thalassemia major population in both genders increases. The explanation for this phenomenon is the coverage of the premarital screening and detection of carrier populations. In other words, the premarital screening includes all about-to-marry couples from all adult classes, whereas the education and marriage reconsideration are conducted only for the part of young adults and carrier populations, respectively. Hence, the absolute magnitude of the sensitivity regarding the premarital screening is biggest among the four parameters in Fig 4. Moreover, since the carrier population will increase as the UAE population grows, the numbers of identified carrier population will rise via the premarital screening. This implies the potential increase of carrier-carrier marriages, which leads to a probable growth of thalassemia major population.

**Fig 5:**
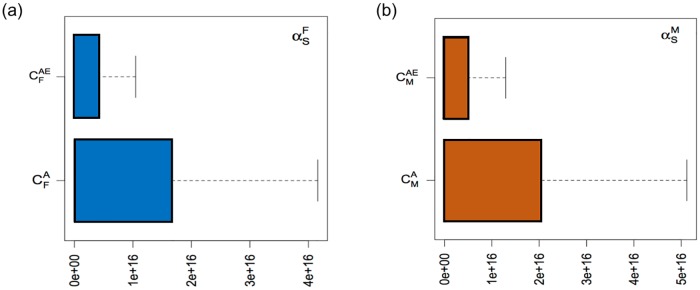
Sensitivity regarding premarital screening rates with respect to educated and uneducated carrier populations. (a) Sensitivity with respect to uneducated and educated carrier single males, $C_{M}^{A}$ and $C_{M}^{AE}.$ (b) Sensitivity with respect to uneducated and educated carrier single females, $C_{F}^{A}$ and $C_{F}^{AE}.$ Note that $\alpha_{s}^{M}$ and $\alpha_{s}^{F}$ are the premarital screening rates for males and females who are about to marry.

In Fig 5, as premarital screening rate increases, the increasing number of identified carrier population is observed. This is a benefit of the premarital screening that could detect carrier population over time. The magnitude of sensitivity regarding premarital screening with respect to uneducated carrier singles is larger than that of educated carrier singles. The reason is that the proportion of educated carrier young adults is smaller than the uneducated carrier young adults. Thus, when these populations are about to marry, the more number of uneducated carrier populations will be identified via premarital screening than that of educated carrier populations.

## Discussion

The assessment of the effectiveness of thalassemia prevention measures used in the UAE should not be done only by relying on the decrease in the number of thalassemia cases. It is important to investigate the extent to which these measures are able to sustain the current situation in the UAE and whether these measures can truly eradicate the disease. In order to answer these questions, we presented a mathematical model at a population level to study the reliability of the education factor and premarital screening as control measures to eradicate thalassemia in the UAE. To our best knowledge, this type of assessment has not been done before and there is no mathematical model that has investigated such a problem for thalassemia or any other blood disorders at a population level.

Our model considered the three different age groups of children, young adults and adults, and imposed an education factor about thalassemia on the young adult group to see the effect on the reduction of marriages between carrier-carrier couples. Also, as the UAE government requires premarital screening, the current mandatory premarital screening of about-to-marry couples was considered as another control factor in our model. We had tested the following government hypotheses about thalassemia prevention:

(H1) The premarital screening will prevent the intermarriage in the carrier populations;
(H2) In addition to premarital screening, if young adults were educated about thalassemia, marriage among the carrier populations will be prevented.

Since it is difficult to directly measure the impact of education and premarital screening on the UAE population, we performed parameter sensitivity analysis of the model with respect to the thalassemia major populations and uneducated and educated carrier populations as shown in Figs 3–5. We found that as birth rates increased, the thalassemia major population increased; and that as marriage reconsideration rates increased, the thalassemia major population decreased. The birth rate effect is due to the rapid population growth of the UAE whereby the thalassemia carrier populations who still marry and could produce thalassemia major children increase. Interestingly, the sensitivity regarding education and premarital screening rates showed two opposite directions. That is, as the rate of education about thalassemia increased, the number of thalassemia cases decreased. On the other hand, as the rate of premarital screening increased, the thalassemia major population tended to increase. As mentioned before, the UAE population growth results in the growth of carrier population. Thus, as the carrier population grows, the premarital screening will identify the increasing number of carrier population, which implies the future growth of thalassemia major children in the UAE. Therefore, the sensitivity result regarding premarital screening measures the depth of the potential problem of carrier-carrier marriages.

Our mathematical analysis and simulations in Fig 2 showed that although these control measures such as education about thalassemia and premarital screening seem to help in reducing the number of thalassemia cases in the the UAE, this decrease in cases is not an indicator of a possible thalassemia-free status in the future. In fact, the number of thalassemia cases will increase eventually. Thus, we summarize what we found from our mathematical modeling as follows:

1. Premarital screening and education about thalassemia do not effectively reduce marriages between carrier populations;
2. Unless all carrier-carrier marriages are stopped, the eradication of thalassemia can not be guaranteed;
3. As long as a carrier population is present in the whole population, a thalassemia major population will always exist;
4. Unless the birth rate is strictly smaller than the death rate of the whole population, thalassemia-free status will never be achieved no matter what control measures are used.

The result of our study regarding premarital screening and carrier-carrier marriages are consistent with the situation in other MENA countries, such as Jordan and the Kingdom of Saudi Arabia, that have a high rate of consanguineous marriage as shown in Table 1. These countries implemented mandatory premarital screening but do not allow termination of pregnancy for thalassemia prevention like the UAE. It has been reported that premarital screening contributed toward detecting carrier population and that carriers who knew their genetic status prior to marriage were more likely to cancel marriage with another carrier in these countries [28]. However, premarital screening did not effectively reduce the carrier-carrier marriages in these countries [28, 29].

The limitation of this work is lack of data on the impact of education about thalassemia and marriage reconsideration of carrier-carrier couples in the UAE. In fact, collecting this type of data, in particular, the impact of education about thalassemia will require the collaboration between local schools and government bodies, valid questionnaires to assess the impacts, and work force to process the collected data. Thus, it may take several years to complete the assessment. The significance of our work is providing a scientific assessment about the impact of education and marriage reconsideration of carrier-carrier couples via mathematical modeling and sensitivity analysis without such data. Furthermore, the result of our study can raise public awareness about the implication of carrier-carrier marriages that have a 1-in-4 chance of having thalassemia major child and the fact that only 100% marriage reconsideration of carrier-carrier couples can lead to the thalassemia eradication in the UAE.

In conclusion, the UAE’s current almost-thalassemia-free status does not imply that the population is approaching true thalassemia free status because there is still a large carrier population in the UAE. The UAE might experience a decrease in thalassemia major population due to government efforts to reduce carrier-carrier marriages, but as soon as the government stops its emphasis on thalassemia control at present level, the thalassemia major population will experience a resurgence. Therefore, we suggest that the UAE government continue its current level of thalassemia management and develop various additional ways to reduce carrier-carrier marriages.

## Supporting Information

S1 Material Supplementary material for mathematical model. (PDF)
