# Supplementary material for: Thalassemia in the United Arab Emirates: Why it can be prevented but not eradicated
Source: PLoS One. 2017 Jan 30;12(1):e0170485. doi: 10.1371/journal.pone.0170485 (PMC5279727; doi:10.1371/journal.pone.0170485)
Supplement: S1 Material — (PDF) [file pone.0170485.s001.pdf]

Supplementary S1 Material to:

"Thalassemia in the United Arab Emirates: Why It Can Be Prevented but Not Eradicated"

Sehjeong Kim[1], Abdessamad Tridane[1]

1 Department of Mathematical Sciences

United Arab Emirates University, Al Ain, Abu Dhabi, P.O. Box 15551, UAE

{sehjung.kim,a-tridane}@uaeu.ac.ae

I. Mathematical Model

The variables and the parameters are given as follows:

Variables

(v1) G_M and G_F are boys and girls (children) under the age of twenty;

(v2) S_M and S_F are the single male and female classes, and C_M and C_F are the single male and female carrier classes. These classes are populations at or over the age of twenty who can be called young adults;

(v3) S_M^A and S_F^A are the marriageable single male and female classes, C_M^A and C_F^A are the marriageable single carrier male and female classes;

(v4) U is the married (or united) class, and T_M and T_F are the male and female thalassemia major classes;

(v5) S_K and S_K^{AE} are uneducated/educated K populations, respectively for $K = M$ or F;

(v6) S_M^{AE}, S_F^{AE}, C_M^{AE}, and C_F^{AE} are educated marriageable male and female singles and carrier populations;

(v7) $N_M = S_M + S_M^E + S_M^{AE} + C_M^E + C_M^{AE}$ and $N_F = S_F + S_F^E + S_F^{AE} + C_F^E + C_F^{AE}$ are denoted as the total adult male and female populations, respectively;

Parameters

(p1) α_M and α_F are marriage rates of male and female;

(p2) α_s^M and α_s^F are the premarital screening rates of single male and female;

(p3) η_T^M and η_T^F are the rates of being diagnosed as thalassemia major of male and female, respectively;

(p4) η_C^M and η_C^F are the rates of being identified as thalassemia carrier of male and female, respectively;

(p5) d_M and d_F are the natural death rates of male and female, and d_T is thalassemia induced death rate;

(p6) b_M and b_F are the birth rates of boys and girls, respectively;

(p7) γ_M and γ_F are the proportions of children becoming young adults;

(p8) ν_M and ν_F are the mariage reconsideration rates of uneducated male and female carrier populations;

(p9) ε is a proportion of educating marriageable single populations;

(p10) $\tilde{\nu}_M$ and $\tilde{\nu}_F$ are marriage reconsideration rates of educated male and female carrier populations;

(p11) d_M^G and d_F^G are child mortality rates.

Then, the full scope of the mathematical model for the thalassemia dynamics with the premarital and education factor is given by

$$\frac{dG_M}{dt} = b_M U - \zeta\eta_T^M G_M - (1-\zeta\eta_T^M)\gamma_M G_M - d_M^G G_M \tag{1}$$

$$\frac{dG_F}{dt} = b_F U - \zeta\eta_T^F G_F - (1-\zeta\eta_T^F)\gamma_F G_F - d_F^G G_F \tag{2}$$

$$\frac{dT_M}{dt} = \zeta\eta_T^M G_M - \left(\frac{\varepsilon(1-\zeta\eta_T^M)\gamma_M G_M}{\gamma_M G_M + \gamma_F G_F}\right) T_M - d_T T_M \tag{3}$$

$$\frac{dT_F}{dt} = \zeta\eta_T^F G_F - \left(\frac{\varepsilon(1-\zeta\eta_T^F)\gamma_F G_F}{\gamma_M G_M + \gamma_F G_F}\right) T_F - d_T T_F \tag{4}$$

$$\frac{dS_M}{dt} = (1-\varepsilon)(1-\zeta\eta_T^M)\gamma_M G_M - \alpha_s^M S_M - d_M S_M \tag{5}$$

$$\frac{dS_M^E}{dt} = \varepsilon(1-\zeta\eta_T^M)\gamma_M G_M - \alpha_s^M S_M^E - d_M S_M^E \tag{6}$$

$$\frac{dS_M^{AE}}{dt} = (1-\eta_C^M)\alpha_s^M S_M^E - \alpha_M S_M^{AE}\frac{(S_F^{AE}+C_F^{AE}+S_F^A+C_F^A)}{N^A} - d_M S_M^{AE} \tag{7}$$

$$\frac{dC_M^{AE}}{dt} = \eta_C^M \alpha_s^M S_M^E - \alpha_M C_M^{AE}\frac{(S_F^{AE}+C_F^{AE}+S_F^A+C_F^A)}{N^A} \tag{8}$$

$$+\tilde{\nu}_M \alpha_M C_M^{AE}\frac{(C_F^A+C_F^{AE})}{N^A} - d_M C_M^{AE} \tag{9}$$

$$\frac{dS_M^A}{dt} = (1-\eta_C^M)\alpha_s^M S_M - \alpha_M S_M^A\frac{(S_F^{AE}+C_F^{AE}+S_F^A+C_F^A)}{N^A} - d_M S_M^A \tag{10}$$

$$\frac{dC_M^A}{dt} = \eta_C^M \alpha_s^M S_M - \alpha_M C_M^A\frac{(S_F^{AE}+C_F^{AE}+S_F^A+C_F^A)}{N^A} \tag{11}$$

$$+\nu_M \alpha_M C_M^A\frac{(C_F^A+C_F^{AE})}{N^A} - d_M C_M^A \tag{12}$$

$$\frac{dS_F}{dt} = (1-\varepsilon)(1-\zeta\eta_T^F)\gamma_F G_F - \alpha_s^F S_F - d_F S_F \tag{13}$$

$$\frac{dS_F^E}{dt} = \varepsilon(1-\zeta\eta_T^F)\gamma_F G_F - \alpha_s^F S_F^E - d_F S_F^E \tag{14}$$

$$\frac{dS_F^{AE}}{dt} = (1-\eta_C^F)\alpha_s^F S_F^E - \alpha_F S_F^{AE}\frac{(S_M^{AE}+C_M^{AE}+S_M^A+C_M^A)}{N^A} - d_F S_F^{AE} \tag{15}$$

$$\frac{dC_F^{AE}}{dt} = \eta_C^F\alpha_s^F S_F^E - \alpha_F C_F^{AE}\frac{(S_M^{AE}+C_M^{AE}+S_M^A+C_M^A)}{N^A} \tag{16}$$

$$+\tilde{\nu}_F\alpha_F C_F^{AE}\frac{(C_M^A+C_M^{AE})}{N^A} - d_F C_F^{AE} \tag{17}$$

$$\frac{dS_F^A}{dt} = (1-\eta_C^F)\alpha_s^F S_F - \alpha_F S_F^A\frac{(S_M^{AE}+C_M^{AE}+S_M^A+C_M^A)}{N^A} - d_F S_F^A \tag{18}$$

$$\frac{dC_F^A}{dt} = \eta_C^F\alpha_s^F S_F - \alpha_F C_F^A\frac{(S_M^{AE}+C_M^{AE}+S_M^A+C_M^A)}{N^A} \tag{19}$$

$$+\nu_F\alpha_F C_F^A\frac{(C_M^A+C_M^{AE})}{N^A} - d_F C_F^A \tag{20}$$

$$\frac{dU}{dt} = \alpha_M(S_M^{AE}+C_M^{AE}+S_M^A+C_M^A)\frac{(S_F^{AE}+C_F^{AE}+S_F^A+C_F^A)}{N^A} \tag{21}$$

$$+\alpha_F(S_F^{AE}+C_F^{AE}+S_F^A+C_F^A)\frac{(S_M^{AE}+C_M^{AE}+S_M^A+C_M^A)}{N^A} \tag{22}$$

$$-\tilde{\nu}_M\alpha_M C_M^{AE}\frac{(C_F^A+C_F^{AE})}{N^A} - \nu_M\alpha_M C_M^A\frac{(C_F^A+C_F^{AE})}{N^A} \tag{23}$$

$$-\tilde{\nu_F}\alpha_F C_F^{AE}\frac{(C_M^A+C_M^{AE})}{N^A} - \nu_F\alpha_F C_F^A\frac{(C_M^A+C_M^{AE})}{N^A} - \frac{1}{2}(d_M+d_F)U, \tag{24}$$

where

$$\zeta = \frac{1}{U}\Bigg(\alpha_M(1-\tilde{\nu}_M)C_M^{AE}\frac{(C_F^{AE}+C_F^A)}{N^A} + \alpha_M(1-\nu_M)C_M^A\frac{(C_F^{AE}+C_F^A)}{N^A}$$

$$+\alpha_F(1-\tilde{\nu}_F)C_F^{AE}\frac{(C_M^{AE}+C_M^A)}{N^A} + \alpha_F(1-\nu_F)C_F^A\frac{(C_M^{AE}+C_M^A)}{N^A}\Bigg)$$

is the proportion of carrier-carrier marriages without marriage reconsideration even with the education and premarital screening.

We provide the details of the model equations in (1) to (24) as follows: Note that all female population will have the similar features as the male population presented here, hence we omit the explanation about the female classes.

- Children Classes (G_M and G_F)

  In Eqs. (1) and (2), $b_M U$ and $b_F U$ are birth of boys and girls, $\zeta\eta_T^M G_M$ and $\zeta\eta_T^F G_F$ are diagnosed thalassemia populations proportional to the carrier-carrier marriages. $(1-\zeta\eta_T^M)\gamma_M G_M$ and $(1-\zeta\eta_T^F)\gamma_F G_F$ are non thalassemia children who become young adults at the age of twenty years old, and $d_M^G G_M$ and $d_F^G G_F$ are death of the children populations.

- Thalassemia Classes (T_M and T_F)

  In Eqs. (3) and (4), $\zeta\eta_T^M G_M$ and $\zeta\eta_T^F G_F$ are diagnosed thalassemia populations and $d_T T_M$ is the death of thalassemia population due to the illness. $-\left(\dfrac{\varepsilon(1-\zeta\eta_T^M)\gamma_M G_M}{\gamma_M G_M+\gamma_F G_F}\right)T_M$ and $-\left(\dfrac{\varepsilon(1-\zeta\eta_T^F)\gamma_F G_F}{\gamma_M G_M+\gamma_F G_F}\right)T_F$ are the reduction of thalassemia populations due to the education of non-thalassemia young adults on thalassemia.

- Single non-educated and educated male classes (S_M and S_M^E)

  In Eqs. (5) and (6), $(1-\varepsilon)(1-\zeta\eta_T^M)\gamma_M G_M$ are non-educated single males who are non-thalassemia and young adults, and $\varepsilon(1-\zeta\eta_T^M)\gamma_M G_M$ are educated single males who are non-thalassemia young adults. $\alpha_s^M S_M$ and $\alpha_s^M S_M^E$ are populations who take the premarital screening when they are about to marry. $d_M S_M$ and $d_M S_{EM}$ are death of the two classes, respectively.

- Single educated marriageable male class (S_M^{AE})

  In Eq. (7), $(1-\eta_C^M)\alpha_s^M S_M^E$ is the normal and non-carrier population screened from the educated single male population, and $\alpha_M S_M^{AE}\dfrac{(S_F^{AE}+C_F^{AE}+S_F^A+C_F^A)}{N^A}$ is the marrying population who will be flushed to the married class U. $d_M S_M^{AE}$ is the death of this class.

- Carrier (single) educated marriageable male class (C_M^{AE})

  In Eqs. (8) and (9), $\eta_C^M\alpha_s^M S_M^E$ is the carrier population screened from the educated single male population, and $\alpha_M C_M^{AE}\dfrac{(S_F^{AE}+C_F^{AE}+S_F^A+C_F^A)}{N^A}$ is the marrying carrier population who will be flushed to the married class U. $\tilde{\nu}_M\alpha_M C_M^{AE}\dfrac{(C_F^A+C_F^{AE})}{N^A}$ is the

proportion of the educated carrier males who **decide not to marry carrier females**. This is the proportion of reconsideration of marriage of carrier male population who have been educated on thalassemia. $d_M C_M^{AE}$ is the death of this class.

- Single and carrier (single) marriageable who are NOT educated on thalassemia (S_M^A and C_M^A)

  In Eqs. (10) and (11), $(1-\eta_C^M)\alpha_s^M S_M$ and $\eta_C^M \alpha_s^M S_M$ are normal and carrier male populations screened from the non educated single male and carrier male populations. The marriages of these populations are $\alpha_M S_M^A \frac{(S_F^{AE}+C_F^{AE}+S_F^A+C_F^A)}{N^A}$ and $\alpha_M C_M^A \frac{(S_F^{AE}+C_F^{AE}+S_F^A+C_F^A)}{N^A}$, respectively. $d_M S_M^A$ and $d_M C_M^A$ are the deaths of these classes, respectively. In Eq. (12) $\nu_M \alpha_M C_M^A \frac{(C_F^A+C_F^{AE})}{N^A}$ is the proportion of the uneducated carrier males who **decide not to marry carrier females**. This is the proportion of reconsideration of marriage of carrier male population who have been screened and knows the consequence of the carrier-carrier marriage.

- Married (united) class (U)

  In Eqs. (21) and (22), $\alpha_M(S_M^{AE}+C_M^{AE}+S_M^A+C_M^A)\frac{(S_F^{AE}+C_F^{AE}+S_F^A+C_F^A)}{N^A}$ and $\alpha_F(S_F^{AE}+C_F^{AE}+S_F^A+C_F^A)\frac{(S_M^{AE}+C_M^{AE}+S_M^A+C_M^A)}{N^A}$ are married males and females, respectively. In Eqs. (23) and (24), $\tilde{\nu}_M \alpha_M C_M^{AE}\frac{(C_F^A+C_F^{AE})}{N^A}$ and $\tilde{\nu_F}\alpha_F C_F^{AE}\frac{(C_M^A+C_M^{AE})}{N^A}$ are the proportion of carrier educated males and females who reconsider their carrier-carrier marriage, and $\nu_M\alpha_M C_M^A\frac{(C_F^A+C_F^{AE})}{N^A}$ and $\nu_F\alpha_F C_F^A\frac{(C_M^A+C_M^{AE})}{N^A}$ are that of carrier non-educated males and females who reconsider their carrier-carrier marriage. Finally, $\frac{(d_M+d_F)U}{2}$ is the death of this class.

II. Analysis of Model

Note that all variables remain nonnegative, i.e. $\frac{dH}{dt}>0$ if $H=0$, where $H=\{G_M, G_F, S_M, S_M^E, S_M^{AE}, C_M^{AE}, S_M^A, C_M^A, S_M, S_F^E, S_F^{AE}, C_F^{AE}, S_F^A, C_F^A, U\}$. Also,

$$\frac{dN}{dt}\le (b_M+b_F)U-\bar{d}N \Rightarrow N(t)\le \frac{b_M+b_F}{\bar{d}}\bar{U},$$

where $N = \sum_{K=M,F}(G_K + S_K^E + S_K^{AE} + C_K^{AE} + S_K^A + C_K^A) + U$, $\bar{U}$ is the maximum number of married couples over a considered time span, and $\bar{d}$ is the minimum of all death rates. Then, we consider two ideal situations that are represented by two equilibrium points, namely,

(i) Type I: Thalassemia free equilibrium point, i.e., $T_M = T_F = 0$, $C_M = C_M^A = C_M^{AE} = 0$, and $C_F = C_F^A = C_F^{AE} = 0$;

(ii) Type II: Thalassemia major free only equilibirium point, i.e., $T_M = T_F = 0$ only C_M, C_M^A, C_M^{AE}, C_F, C_F^A, C_F^{AE} are not necessarily zero

as time evolves in a long term. To investigate if (i) and (ii) are achievable via pre-marital screening and education factor, we will calculate two jacobian matrices which are obtained by differentiating each equation in eqs. (1) to (24) with seventeen variables in order $(G_M, G_F, T_M, T_F, S_M, S_M^E, S_M^{AE}, C_M^{AE}, S_M^A, C_M^A, S_F, S_F^E, S_F^{AE}, C_F^{AE}, S_F^A, C_F^A, U)$ and substituting the two equilibrium points in them.

Type I. Thalassemia free equlibrium point
$(G_M^*, G_F^*, 0, 0, S_M^*, S_M^{AE^*}, 0, S_M^{A^*}, 0, S_F^*, S_F^{AE^*}, 0, S_F^{A^*}, 0, U^*)$

For this equilibrium point, we obtain the following jacobian matrix J_1:

$$\begin{bmatrix}
-a_1 & 0 & 0 & 0 & 0 & 0 & 0 & 0 & 0 & 0 & 0 & 0 & 0 & 0 & 0 & 0 & b_M \\
0 & -b_2 & 0 & 0 & 0 & 0 & 0 & 0 & 0 & 0 & 0 & 0 & 0 & 0 & 0 & 0 & b_F \\
0 & 0 & -c_3 & 0 & 0 & 0 & 0 & 0 & 0 & 0 & 0 & 0 & 0 & 0 & 0 & 0 & 0 \\
0 & 0 & 0 & -d_4 & 0 & 0 & 0 & 0 & 0 & 0 & 0 & 0 & 0 & 0 & 0 & 0 & 0 \\
e_1 & 0 & 0 & 0 & -e_5 & 0 & 0 & 0 & 0 & 0 & 0 & 0 & 0 & 0 & 0 & 0 & 0 \\
f_1 & 0 & 0 & 0 & 0 & -f_6 & 0 & 0 & 0 & 0 & 0 & 0 & 0 & 0 & 0 & 0 & 0 \\
0 & 0 & 0 & 0 & 0 & g_6 & -B & D & D & D & 0 & 0 & -D & -D & -D & -D & 0 \\
0 & 0 & 0 & 0 & 0 & h_6 & 0 & -C & 0 & 0 & 0 & 0 & 0 & 0 & 0 & 0 & 0 \\
0 & 0 & 0 & 0 & i_5 & 0 & E & E & -\tilde{B} & E & 0 & 0 & -E & -E & -E & -E & 0 \\
0 & 0 & 0 & 0 & 0 & 0 & 0 & 0 & 0 & -C & 0 & 0 & 0 & 0 & 0 & 0 & 0 \\
0 & k_2 & 0 & 0 & 0 & 0 & 0 & 0 & 0 & 0 & -k_{11} & 0 & 0 & 0 & 0 & 0 & 0 \\
0 & l_2 & 0 & 0 & 0 & 0 & 0 & 0 & 0 & 0 & 0 & -l_{12} & 0 & 0 & 0 & 0 & 0 \\
0 & 0 & 0 & 0 & 0 & 0 & -H & -H & -H & -H & 0 & m_{12} & -F & H & H & H & 0 \\
0 & 0 & 0 & 0 & 0 & 0 & 0 & 0 & 0 & 0 & 0 & n_{12} & 0 & -G & 0 & 0 & 0 \\
0 & 0 & 0 & 0 & 0 & 0 & -I & -I & -I & -I & p_{11} & 0 & I & I & -\tilde{F} & I & 0 \\
0 & 0 & 0 & 0 & 0 & 0 & 0 & 0 & 0 & 0 & q_{11} & 0 & 0 & 0 & 0 & -G & 0 \\
0 & 0 & 0 & 0 & 0 & 0 & J & J & J & J & 0 & 0 & \tilde{J} & \tilde{J} & \tilde{J} & \tilde{J} & r_{17}
\end{bmatrix}, \tag{25}$$

where

- $a_1 = d_M^G + \gamma_M$, $b_2 = d_F^G + \gamma_F$, $c_3 = d_T + \dfrac{\varepsilon\gamma_M G_M^*}{\gamma_M G_M^* + \gamma_F G_F^*}$ and $d_4 = d_T + \dfrac{\varepsilon\gamma_F G_F^*}{\gamma_M G_M^* + \gamma_F G_F^*}$;
- $e_1 = \gamma_M(1-\varepsilon)$ and $e_5 = \alpha_s^M + d_M$, and $f_1 = \varepsilon\gamma_M$ and $f_6 = \alpha_s^M + d_M$;
- $g_6 = \alpha_s^M(1-\eta_M^C)$, $B = d_M + \alpha_M N_{AF}^* \dfrac{(N_A^* - S_M^{AE^*})}{(N_A^*)^2}$ and $D = \dfrac{\alpha_M N_{AF}^* - S_M^{AE^*}}{(N_A^*)^2}$, where $N_A^* = N_{AM}^* + N_{AF}^*$, $N_{AM}^* = S_M^{AE^*} + S_M^{A^*}$ and $N_{AF}^* = S_F^{AE^*} + S_F^{A^*}$;
- $h_6 = \alpha_s^M \eta_M^C$ and $C = d_M + \dfrac{\alpha_M N_{AF}^*}{N_A^*}$;
- $i_5 = \alpha_s^M$, $\tilde{B} = d_M + \alpha_M N_{AF}^* \dfrac{(N_A^* - S_M^{A^*})}{(N_A^*)^2}$, and $E = \dfrac{\alpha_M N_{AF}^* S_M^{A^*}}{(N_A^*)^2}$;
- $k_2 = \gamma_F(1-\varepsilon)$ and $k_{11} = \alpha_s^F + d_F$, $l_2 = \varepsilon\gamma_F$ and $l_{12} = \alpha_s^F + d_F$;
- $m_{12} = \alpha_s^F(1-\eta_F^C)$, $F = d_F + \alpha_F N_{AM}^* \dfrac{(N_A^* - S_F^{AE^*})}{(N_A^*)^2}$, and $H = \dfrac{\alpha_F N_{AM}^* S_F^{AE^*}}{(N_A^*)^2}$;

- $n_{12} = \alpha_s^F \eta_F^C$ and $G = d_F + \dfrac{\alpha_F N_{AM}^*}{N_A^*}$;

- $p_{11} = \alpha_s^F(1 - \eta_F^C)$, $\tilde{F} = d_F + \alpha_F N_{AM}^* \dfrac{(N_A^* - S_F^{A^*})}{(N_A^*)^2}$, and $I = \dfrac{\alpha_F N_{AM}^* S_F^{A^*}}{(N_A^*)^2}$;

- $q_{11} = \alpha_s^F \eta_F^C$, $r_{17} = -\dfrac{d_M + d_F}{2}$, $J = \dfrac{(\alpha_M + \alpha_F)(N_{AF}^*)^2}{(N_A^*)^2}$, and $\tilde{J} = \dfrac{(\alpha_M + \alpha_F)(N_{AM}^*)^2}{(N_A^*)^2}$.

Type II. Thalassemia major free only equilibrium point
$(G_M^*, G_F^*, 0, 0, S_M^*, S_M^{AE^*}, C_M^{AE^*}, S_M^{A^*}, C_M^{A^*}, S_F^*, S_F^{AE^*}, C_F^{AE^*}, S_F^{A^*}, C_F^{A^*}, U^*)$

For this equilibrium point we obtain the following jacobian matrix J_2 :

$$\begin{bmatrix}
-a_1 & 0 & 0 & 0 & 0 & 0 & A & A_t & A & C_t & 0 & 0 & A & A'_t & A & C'_t & a_{17} \\
0 & -b_2 & 0 & 0 & 0 & 0 & \tilde{A} & \tilde{A}_t & \tilde{A} & \tilde{C}_t & 0 & 0 & \tilde{A} & \tilde{A'}_t & \tilde{A} & \tilde{C'}_t & b_{17} \\
c_1 & 0 & -c_3 & 0 & 0 & 0 & -B & -B_t & -B & -D_t & 0 & 0 & -B & -B'_t & -B & -D'_t & c_{17} \\
0 & d_2 & 0 & -d_4 & 0 & 0 & -\tilde{B} & -\tilde{B}_t & -\tilde{B} & -\tilde{D}_t & 0 & 0 & -\tilde{B} & -\tilde{B'}_t & -\tilde{B} & -\tilde{D'}_t & d_{17} \\
e_1 & 0 & 0 & 0 & -e_5 & 0 & E & E_t & E & F_t & 0 & 0 & E & E'_t & E & F'_t & -e_{17} \\
f_1 & 0 & 0 & 0 & 0 & -f_6 & \tilde{E} & \tilde{E}_t & \tilde{E} & \tilde{F}_t & 0 & 0 & \tilde{E} & \tilde{E'}_t & \tilde{E} & \tilde{F'}_t & -f_{17} \\
0 & 0 & 0 & 0 & 0 & g_6 & -g_7 & G & G & G & 0 & 0 & -\tilde{M} & -\tilde{M} & -\tilde{M} & -\tilde{M} & 0 \\
0 & 0 & 0 & 0 & 0 & h_6 & H & -h_8 & H & H & 0 & 0 & -\tilde{N} & -\tilde{P} & -\tilde{N} & -\tilde{P} & 0 \\
0 & 0 & 0 & 0 & i_5 & 0 & I & I & -i_9 & I & 0 & 0 & -\tilde{Q} & -\tilde{Q} & -\tilde{Q} & -\tilde{Q} & 0 \\
0 & 0 & 0 & 0 & j_5 & 0 & J & J & J & -j_{10} & 0 & 0 & -\tilde{R} & -\tilde{S} & -\tilde{R} & -\tilde{S} & 0 \\
0 & k_2 & 0 & 0 & 0 & 0 & K & L_t & K & L & -k_{11} & 0 & K & L'_t & K & L' & -k_{17} \\
0 & l_2 & 0 & 0 & 0 & 0 & \tilde{K} & \tilde{L}_t & \tilde{K} & \tilde{L} & 0 & -l_{12} & \tilde{K} & \tilde{L'}_t & \tilde{K} & \tilde{L'} & -l_{17} \\
0 & 0 & 0 & 0 & 0 & 0 & -M & -M & -M & -M & 0 & m_{12} & -m_{13} & \tilde{G} & \tilde{G} & \tilde{G} & 0 \\
0 & 0 & 0 & 0 & 0 & 0 & -N & -P & -N & -P & 0 & n_{12} & \tilde{H} & -n_{14} & \tilde{H} & \tilde{H} & 0 \\
0 & 0 & 0 & 0 & 0 & 0 & -Q & -Q & -Q & -Q & p_{11} & 0 & \tilde{I} & \tilde{I} & -p_{15} & \tilde{I} & 0 \\
0 & 0 & 0 & 0 & 0 & 0 & -R & -S & -R & -S & q_{11} & 0 & \tilde{J} & \tilde{J} & \tilde{J} & -q_{16} & 0 \\
0 & 0 & 0 & 0 & 0 & 0 & X & Y & X & Y & 0 & 0 & X & \tilde{Y} & X & \tilde{Y} & -r_{17}
\end{bmatrix}, \tag{26}$$

where

(i) $C_{Fadd} = (C_F^{A^*} + C_F^{AE^*})$ and $C_{Madd} = (C_M^{A^*} + C_M^{AE^*})$
$C_{FaddnuM} = \alpha_M (C_F^{A^*} + C_F^{AE^*})\{(1 - \tilde{\nu}_M) C_M^{AE^*} + (1 - \nu_M) C_M^{A^*}\}$
$C_{MaddnuF} = \alpha_F (C_M^{A^*} + C_M^{AE^*})\{(1 - \tilde{\nu}_F) C_F^{AE^*} + (1 - \nu_F) C_F^{A^*}\}$
$N_{AF}^* = S_M^{AE^*} + S_M^{A^*} + C_M^{AE^*} + C_M^{A^*}$ and $N_{AM}^* = S_F^{AE^*} + S_F^{A^*} + C_F^{AE^*} + C_F^{A^*}$
$N_A^* = N_{AF}^* + N_{AM}^*$;

(ii) $Z_1 = \dfrac{C_{FaddnuM} + C_{MaddnuF}}{N_A^*}$;

(iii) $\tilde{Z}_2 = \dfrac{\alpha_F((1-\nu_F)C_F^{A^*} + (1-\tilde{\nu}_F)C_F^{AE^*}) + \alpha_M(1-\tilde{\nu}_M)C_{Fadd}}{N_A^*}$;

(iv) $Z_2 = \dfrac{\alpha_F((1-\nu_F)C_F^{A^*} + (1-\tilde{\nu}_F)C_F^{AE^*}) + \alpha_M(1-\nu_M)C_{Fadd}}{N_A^*}$;

(v) $\tilde{Z}_3 = \dfrac{\alpha_M((1-\nu_M)C_M^{A^*} + (1-\tilde{\nu}_M)C_M^{AE^*}) + \alpha_F(1-\tilde{\nu}_F)C_{Madd}}{N_A^*}$;

(vi) $Z_3 = \dfrac{\alpha_M((1-\nu_M)C_M^{A^*} + (1-\tilde{\nu}_M)C_M^{AE^*}) + \alpha_F(1-\nu_F)C_{Madd}}{N_A^*}$;

- $a_1 = d_M^G + \gamma_M + \dfrac{(1-\gamma_M)\eta_M^T Z_1}{U^*}$, $c_1 = \dfrac{\eta_M^T Z_1}{U^*}$, $e_1 = \gamma_M(1-\varepsilon)(1-\eta_M^T)\dfrac{Z_1}{U^*}$ and $f_1 = \gamma_M\varepsilon(1-\eta_M^T)\dfrac{Z_1}{U^*}$;
- $b_2 = d_F^G + \gamma_F + \dfrac{(1-\gamma_F)\eta_F^T Z_1}{U^*}$, $d_2 = \dfrac{\eta_F^T Z_1}{U^*}$, $k_2 = \gamma_F(1-\varepsilon)(1-\eta_F^T)\dfrac{Z_1}{U^*}$ and $l_2 = \gamma_F\varepsilon(1-\eta_F^T)\dfrac{Z_1}{U^*}$;
- $c_3 = d_T + \dfrac{\varepsilon\gamma_M(1-\eta_M^T)G_M Z_1}{(\gamma_F G_F + \gamma_M G_M)U^*}$ and $d_4 = d_T + \dfrac{\varepsilon\gamma_F(1-\eta_F^T)G_F Z_1}{(\gamma_F G_F + \gamma_M G_M)U^*}$;
- $e_5 = f_6 = d_M + \alpha_s^M$, $i_5 = g_6 = \alpha_s^M(1-\eta_M^T)$, and $j_5 = h_6 = \alpha_s^M\eta_M^T$;
- $g_7 = d_M + \dfrac{\alpha_M N_{AF}^*(N_A^* - S_M^{AE^*})}{(N_A^*)^2}$ and $h_8 = d_M + \dfrac{\alpha_M(N_A^* - C_M^{AE^*})(N_{AF}^* - \tilde{\nu}_M C_{Fadd})}{(N_A^*)^2}$, $i_9 = d_M + \dfrac{\alpha_M N_{AF}^*(N_A^* - S_M^{A^*})}{(N_A^*)^2}$ and $j_{10} = d_M + \dfrac{\alpha_M(N_A^* - C_M^{A^*})(N_{AF}^* - \nu_M C_{Fadd})}{(N_A^*)^2}$;
- $k_{11} = l_{12} = d_F + \alpha_s^F$, $p_{11} = m_{12} = \alpha_S^F(1-\eta_F^C)$, and $q_{11} = n_{12} = \alpha_s^F\eta_F^C$;
- $m_{13} = d_F + \dfrac{\alpha_F N_{AM}^*(N_A^* - S_F^{AE^*})}{(N_A^*)^2}$ and $n_{14} = d_F + \dfrac{\alpha_F(N_A^* - C_F^{AE^*})(N_{AM}^* - \tilde{\nu}_F C_{Madd})}{(N_A^*)^2}$, $p_{15} = d_F + \dfrac{\alpha_F N_{AM}^*(N_A^* - S_F^{A^*})}{(N_A^*)^2}$ and $q_{16} = d_F + \dfrac{\alpha_F(N_A^* - C_F^{A^*})(N_{AM}^* - \nu_F C_{Madd})}{(N_A^*)^2}$;
- $a_{17} = b_M - \dfrac{(1-\gamma_M)\eta_M^T G_M^* Z_1}{(U^*)^2}$ and $b_{17} = b_F - \dfrac{(1-\gamma_F)\eta_F^T G_F^* Z_1}{(U^*)^2}$,
  $c_{17} = \dfrac{\eta_M^T G_M^* Z_1}{(U^*)^2}$ and $d_{17} = \dfrac{\eta_F^T G_F^* Z_1}{(U^*)^2}$,
  $e_{17} = \dfrac{(1-\varepsilon)\eta_M^T\gamma_M G_M^* Z_1}{(U^*)^2}$ and $f_{17} = \dfrac{\varepsilon\eta_M^T\gamma_M G_M^* Z_1}{(U^*)^2}$,

$k_{17} = \dfrac{(1-\varepsilon)\eta_F^T \gamma_F G_F^* Z_1}{(U^*)^2}$ and $l_{17} = \dfrac{\varepsilon \eta_F^T \gamma_F G_F^* Z_1}{(U^*)^2}$,
$r_{17} = \dfrac{d_M + d_F}{2}$;

- $A = \dfrac{(1-\gamma_M)\eta_M^T G_M Z_1}{N_A^* U^*}$ and $\tilde{A} = \dfrac{(1-\gamma_F)\eta_F^T G_F Z_1}{N_A^* U^*}$,
  $A_t = \dfrac{(1-\gamma_M)\eta_M^T G_M}{N_A^* U}(Z_1 - \tilde{Z}_2)$ and $\tilde{A}_t = \dfrac{(1-\gamma_F)\eta_F^T G_F}{N_A^* U^*}(Z_1 - \tilde{Z}_2)$,
  $A_t' = \dfrac{(1-\gamma_M)\eta_M^T G_M}{N_A^* U^*}(Z_1 - \tilde{Z}_3)$ and $\tilde{A}_t' = \dfrac{(1-\gamma_F)\eta_F^T G_F}{N_A^* U^*}(Z_1 - \tilde{Z}_3)$
  $C_t = \dfrac{(1-\gamma_M)\eta_M^T G_M}{N_A^* U^*}(Z_1 - Z_2)$ and $\tilde{C}_t = \dfrac{(1-\gamma_F)\eta_F^T G_F}{N_A^* U^*}(Z_1 - Z_2)$,
  $C_t' = \dfrac{(1-\gamma_M)\eta_M^T G_M}{N_A^* U^*}(Z_1 - Z_3)$ and $\tilde{C}_t' = \dfrac{(1-\gamma_F)\eta_F^T G_F}{N_A^* U^*}(Z_1 - Z_3)$;

- $B = \dfrac{\eta_M^T G_M Z_1}{N_A^* U^*}$ and $\tilde{B} = \dfrac{\eta_F^T G_F Z_1}{N_A^* U^*}$,
  $B_t = \dfrac{\eta_M^T G_M}{N_A^* U^*}(Z_1 - \tilde{Z}_2)$ and $\tilde{B}_t = \dfrac{\eta_F^T G_F}{N_A^* U^*}(Z_1 - \tilde{Z}_2)$,
  $B_t' = \dfrac{\eta_M^T G_M}{N_A^* U^*}(Z_1 - \tilde{Z}_3)$ and $\tilde{B}_t' = \dfrac{\eta_F^T G_F}{N_A^* U^*}(Z_1 - \tilde{Z}_3)$,
  $D_t = \dfrac{\eta_M^T G_M}{N_A^* U^*}(Z_1 - Z_2)$ and $\tilde{D}_t = \dfrac{\eta_F^T G_F}{N_A^* U^*}(Z_1 - Z_2)$,
  $D_t' = \dfrac{\eta_M^T G_M}{N_A^* U^*}(Z_1 - Z_3)$ and $\tilde{D}_t' = \dfrac{\eta_F^T G_F}{N_A^* U^*}(Z_1 - Z_3)$;

- $E = \dfrac{(1-\varepsilon)\eta_M^T G_M Z_1}{N_A^* U^*}$, and $\tilde{E} = \dfrac{\varepsilon \eta_M^T G_M Z_1}{N_A^* U^*}$,
  $E_t = \dfrac{(1-\varepsilon)\eta_M^T G_M}{N_A^* U^*}(Z_1 - \tilde{Z}_2)$ and $\tilde{E}_t = \dfrac{\varepsilon \eta_M^T G_M}{N_A^* U^*}(Z_1 - \tilde{Z}_2)$,
  $E_t' = \dfrac{(1-\varepsilon)\eta_M^T G_M}{N_A^* U^*}(Z_1 - \tilde{Z}_3)$ and $\tilde{E}_t' = \dfrac{\varepsilon \eta_M^T G_M}{N_A^* U^*}(Z_1 - \tilde{Z}_3)$,
  $F_t = \dfrac{(1-\varepsilon)\eta_M^T G_M}{N_A^* U^*}(Z_1 - Z_2)$ and $\tilde{F}_t = \dfrac{\varepsilon \eta_M^T G_M}{N_A^* U*}(Z_1 - Z_2)$,
  $F_t' = \dfrac{(1-\varepsilon)\eta_M^T G_M}{N_A^* U^*}(Z_1 - Z_3)$ and $\tilde{F}_t' = \dfrac{\varepsilon \eta_M^T G_M}{N_A^* U^*}(Z_1 - Z_3)$;

- $K = \dfrac{(1-\varepsilon)\eta_F^T \gamma_F G_F Z_1}{N_A^* U^*}$, and $\tilde{K} = \dfrac{\varepsilon \eta_F^T \gamma_F G_F Z_1}{N_A^* U^*}$,
  $L_t = \dfrac{(1-\varepsilon)\eta_F^T \gamma_F G_F}{N_A^* U^*}(Z_1 - \tilde{Z}_2)$ and $\tilde{L}_t = \dfrac{\varepsilon \eta_F^T \gamma_F G_F}{N_A^* U^*}(Z_1 - \tilde{Z}_2)$,
  $L_t' = \dfrac{(1-\varepsilon)\gamma_F \eta_F^T G_F}{N_A^* U^*}(Z_1 - \tilde{Z}_3)$ and $\tilde{L}_t' = \dfrac{\varepsilon \eta_F^T \gamma_F G_F}{N_A^* U^*}(Z_1 - \tilde{Z}_3)$,

$$L = \frac{(1-\varepsilon)\eta_F^T \gamma_F G_F}{N_A^* U^*}(Z_1 - Z_2) \text{ and } \tilde{L} = \frac{\varepsilon \eta_F^T \gamma_F G_F}{N_A^* U^*}(Z_1 - Z_2),$$
$$L' = \frac{(1-\varepsilon)\gamma_F \eta_F^T G_F}{N_A^* U^*}(Z_1 - Z_3) \text{ and } \tilde{L}' = \frac{\varepsilon \eta_F^T \gamma_F G_F}{N_A^* U^*}(Z_1 - Z_3),$$

- $G = \dfrac{\alpha_M S_M^{AE^*} N_{AF}^*}{(N_A^*)^2}$ and $\tilde{G} = \dfrac{\alpha_F S_F^{AE^*} N_{AM}^*}{(N_A^*)^2}$, $I = \dfrac{\alpha_M S_M^{A^*} N_{AF}^*}{(N_A^*)^2}$ and $\tilde{I} = \dfrac{\alpha_F S_F^{A^*} N_{AM}^*}{(N_A^*)^2}$,
$H = \dfrac{\alpha_M C_M^{AE^*}(N_{AF}^* - \tilde{\nu}_M C_{Fadd})}{(N_A^*)^2}$ and $\tilde{H} = \dfrac{\alpha_F C_F^{AE^*}(N_{AM}^* - \tilde{\nu}_F C_{Madd})}{(N_A^*)^2}$,
$J = \dfrac{\alpha_M C_M^{A^*}(N_{AF}^* - \nu_M C_{Fadd})}{(N_A^*)^2}$ and $\tilde{J} = \dfrac{\alpha_F C_F^{A^*}(N_{AM}^* - \nu_F C_{Madd})}{(N_A^*)^2}$,
$P = \dfrac{\alpha_F C_F^{AE^*}(N_{AF}^* - \tilde{\nu}_F(N_A^* - C_{Madd}))}{(N_A^*)^2}$ and $\tilde{P} = \dfrac{\alpha_M C_M^{AE^*}(N_{AM}^* - \tilde{\nu}_M(N_A^* - C_{Fadd}))}{(N_A^*)^2}$,
$S = \dfrac{\alpha_F C_F^{A^*}(N_{AF}^* - \nu_F(N_A^* - C_{Madd}))}{(N_A^*)^2}$ and $\tilde{S} = \dfrac{\alpha_M C_M^{A^*}(N_{AM}^* - \nu_M(N_A^* - C_{Fadd}))}{(N_A^*)^2}$;

- $M = \dfrac{\alpha_F S_F^{AE^*} N_{AF}^*}{(N_A^*)^2}$ and $\tilde{M} = \dfrac{\alpha_M S_M^{AE^*} N_{AM}^*}{(N_A^*)^2}$, $Q = \dfrac{\alpha_F S_F^{A^*} N_{AF}^*}{(N_A^*)^2}$ and $\tilde{Q} = \dfrac{\alpha_M S_M^{A^*} N_{AM}^*}{(N_A^*)^2}$,
$N = \dfrac{\alpha_F C_F^{AE^*}(N_{AF}^* + \tilde{\nu}_F C_{Madd})}{(N_A^*)^2}$ and $\tilde{N} = \dfrac{\alpha_M C_M^{AE^*}(N_{AM}^* + \tilde{\nu}_M C_{Fadd})}{(N_A^*)^2}$,
$R = \dfrac{\alpha_F C_F^{A^*}(N_{AF}^* + \nu_F C_{Madd})}{(N_A^*)^2}$ and $\tilde{R} = \dfrac{\alpha_M C_M^{A^*}(N_{AM}^* + \nu_M C_{Fadd})}{(N_A^*)^2}$,

- $X = \dfrac{(\alpha_M + \alpha_F)(N_{AF^*})^2}{(N_A^*)^2}$
$\qquad \dfrac{+\alpha_F C_{Madd}(\nu_F C_F^{A^*} + \tilde{\nu}_F C_F^{AE^*}) + \alpha_M C_{Fadd}(\nu_M C_M^{A^*} + \tilde{\nu}_M C_M^{AE^*})}{(N_A^*)^2}$,
$Y = X - \dfrac{\alpha_F(\nu_F C_F^{A^*} + \tilde{\nu}_F C_F^{AE^*}) + \alpha_M \tilde{\nu}_M C_{Fadd}}{N_A^*}$,
$\tilde{Y} = X - \dfrac{\alpha_M(\nu_M C_M^{A^*} + \tilde{\nu}_M C_M^{AE^*}) + \alpha_F \tilde{\nu}_F C_{Madd}}{N_A^*}$.

Proposition 1 *[1] [Chapter 6 Corollary 6.1.3] The eigenvalues of $A = [m_{ij}]_{n\times n}$, an n by n matrix, are in the union of n discs*

$$\bigcup_{j=1}^{n} \{z \in \mathbb{C} : |z - a_{jj}| \le C_j'(A)\}, \tag{27}$$

where $\mathbb{C}$ is the set of complex numbers, a_{jj} are the diagonal entries in A, $\{z \in \mathbb{C} : |z - a_{jj}| \le C_j'(A)\}$, is a disc in $\mathbb{C}$ centered at a_{jj} with the radius $C_j'(A)$, and

$$C_j'(A) = \sum_{i\ne j} |a_{ij}|, \ j = 1, \cdots, n, \tag{28}$$

which is the absolute column sum without the the diagonal entry in the jth column.

The above proposition is known as *Geršgorin disc theorem.* By using Proposition 1, we have the following result:

Theorem 2 *Type I equilibrium point, the thalassemia free equilibrium point*

$$(G_M^*, G_F^*, 0, 0, S_M^*, S_M^{AE^*}, 0, S_M^{A^*}, 0, S_F^*, S_F^{AE^*}, 0, S_F^{A^*}, 0, U^*),$$

and Type II equilibrium point, the thalassemia major free only equilibrium point

$$(G_M^*, G_F^*, 0, 0, S_M^*, S_M^{AE^*}, C_M^{AE^*}, S_M^{A^*}, C_M^{A^*}, S_F^*, S_F^{AE^*}, C_F^{AE^*}, S_F^{A^*}, C_F^{A^*}, U^*),$$

are unstable.

Proof From the jacobian matrix J_1, we can calculate $C_j'(J_1) = \sum_{i \neq j} |a_{ij}|, \; j = 1, \cdots, n,$. Then, we have

$$\bigcup_{j=1}^{17} \{z \in \mathbb{C} : |z - a_{jj}| \leq C_j'(J_1)\}, \tag{29}$$

where the discs from $j = 1, \cdots, 6$, and $j = 10, 11$ locate in the left half plane of $\mathbb{C}$ since $|a_{jj}| > C_j'(J_1)$ for such j. However, the rest of discs may cross the origin and the right half plane of $\mathbb{C}$. Note that the 17th disc is given by

$$\{z \in \mathbb{C} : |z - (-\frac{d_M + d_F}{2})| \leq b_M + b_F\} \tag{30}$$

which is the disc centered at $-\dfrac{d_M + d_F}{2}$ with the radius $C_{17}'(J_1) = b_M + b_F$. Since the UAE population is in the increasing trend, i.e. the birth rate is greater than the averaged death rate, we conclude

$$\frac{d_M + d_F}{2} < b_M + b_F. \tag{31}$$

Thus, the 17th disc in (30) surely crosses the origin and the right half plane of $\mathbb{C}$. Thus, Type I equilibrium point is unstable. By the similar argument, from the jacobian matrix J_2 we can calculate $C_j'(J_1) = \sum_{i \neq j} |a_{ij}|, \; j = 1, \cdots, n,$. Then, we have

$$\bigcup_{j=1}^{17} \{z \in \mathbb{C} : |z - a_{jj}| \leq C_j'(J_2)\}, \tag{32}$$

where the discs from $j = 1, \cdots, 6$, and $j = 10, 11$ locate in the left half plane of $\mathbb{C}$ since $|a_{jj}| > C'_j(J_2)$ for such j. However, the rest of discs may cross the origin and the right half plane of $\mathbb{C}$. In particular, the 17th disc is given by

$$\{z \in \mathbb{C} : |z - (-\frac{d_M + d_F}{2})| \leq b_M + b_F + \frac{(\eta_M^T \gamma_M G_M^* + \eta_F^T \gamma_F G_F^*) Z_1}{(U^*)^2}\} \tag{33}$$

which is the disc centered at $-\frac{d_M + d_F}{2}$ with the radius
$C'_{17}(J_2) = b_M + b_F + \frac{(\eta_M^T \gamma_M G_M^* + \eta_F^T \gamma_F G_F^*) Z_1}{(U^*)^2}$, where $Z_1 = \frac{C_{FaddnuM} + C_{MaddnuF}}{N_A^*}$,
$C_{FaddnuM} = \alpha_M (C_F^{A^*} + C_F^{AE^*})\{(1 - \tilde{\nu}_M) C_M^{AE^*} + (1 - \nu_M) C_M^{A^*}\}$, and
$C_{MaddnuF} = \alpha_F (C_M^{A^*} + C_M^{AE^*})\{(1 - \tilde{\nu}_F) C_F^{AE^*} + (1 - \nu_F) C_F^{A^*}\}$. Since the population growth of UAE is in the increasing trend, the birth rates are greater than the death rates and hence

$$\frac{d_M + d_F}{2} < b_M + b_F < b_M + b_F + \frac{(\eta_M^T \gamma_M G_M^* + \eta_F^T \gamma_F G_F^*) Z_1}{(U^*)^2} = C'_{17}(J_2). \tag{34}$$

Thus, the 17th disc surely crosses the origin and hence the right half plane of $\mathbb{C}$. Thus, Type II equilibrium point is unstable. This completes the proof. ∎

Remark In the result in Theorem 2 we observed the 17th disc crosses through the origin from the left half plane to the right half plane of $\mathbb{C}$ from the relation between the birth and death rates of the whole population. Thus, Types I and II equilibrium points are unstable regardless of the premarital screening and education factor. Hence, Types I and II equilibrium points that are our current goal in thalassemia control will not be achievable with the premarital and education factor in a long term.

III. Parameter Estimation

We obtain and estimate the parameter values for the simulations from the 2015 data of the UAE bureau of statistics [2], 2013 Abu Dhabi Statistics Yearbook [3] and 2013 Health Authority of Abh Dhabi [4]. The part of the UAE national population data is summarized in Table 1.

Then, the parameter values are estimated as follows:

- Birth rates: $b_M = \frac{17,279}{476,712} = 0.0362$ and $b_F = \frac{16,761}{476,712} = 0.0352$.

Table 1: **The UAE National Census Data**

Population (2012)			Birth (2012)			Marriage Contracts (2014)		
M	F	Total	M	F	Total	M	F	Total
231,383	245,329	476,712	17,279	16,761	34,040	9560	8239	17,799

Death rates: $d_M = \frac{1432}{476,712} = 0.0030$ and $d_F = \frac{910}{476,712} = 0.0019$

- Marriage rates:
  $\alpha_M = \frac{\text{Male Marriage Contracts}}{\text{Total Population}} = \frac{9560}{476712} = 0.02$

  $\alpha_F = \frac{\text{Female Marriage Contracts}}{\text{Total population}} = \frac{8239}{476712} = 0.0172$

- Screening rates:
  $\alpha_s^M = \frac{\text{Male Marriage Contracts}}{\text{Male total population}} = \frac{9560}{231383} = 0.0413$

  $\alpha_s^F = \frac{\text{Female Marriage Contracts}}{\text{Female total Population}} = \frac{8239}{245329} = 0.0335$

- Thalassemia diagnosis adjusting factor: $\eta_M^T = 0.015$ and $\eta_F^T = 0.144$ are chosen such that the proportion of thalassemia major male is less than 0.00025 and the proportion of thalassemia major female is than 0.00024.

References

[1] Horn R, Johnson C. Matrix analysis. Cambridge University Press; 2012.

[2] Federal Competitiveness and Statistics Authority. Statistics by subjects. 2015. Available from: `http://fcsa.gov.ae`

[3] Statistics Center Abu Dhabi. Statistical yearbook of Abu Dhabi 2013. Government.ae. Available from: `http://government.ae/documents/10138/3525509/SYB+2013+English+-full+version.pdf/324407c4-8e0d-4d09-abab-6983547234c8;jsessionid=23GI0V7f+yzLK2zWOB7DJQgI.undefined`

[4] Health Authority Abu Dhabi. Health Statistics 2013. Haad.ae. 2013. Available from: `http://www.haad.ae`
